# The Pathogenetic Dilemma of Post-COVID-19 Mucormycosis in India

**DOI:** 10.14336/AD.2021.0811

**Published:** 2022-02-01

**Authors:** Sankha Shubhra Chakrabarti, Upinder Kaur, Sushil Kumar Aggarwal, Ahalya Kanakan, Adesh Saini, Bimal Kumar Agrawal, Kunlin Jin, Sasanka Chakrabarti

**Affiliations:** ^1^Department of Geriatric Medicine, Institute of Medical Sciences, Banaras Hindu University, Varanasi, UP, India.; ^2^Department of Pharmacology, Institute of Medical Sciences, Banaras Hindu University, Varanasi, UP, India.; ^3^Department of Otorhinolaryngology, Institute of Medical Sciences, Banaras Hindu University, Varanasi, UP, India.; ^4^Department of Geriatric Medicine, Institute of Medical Sciences, Banaras Hindu University, Varanasi, UP, India.; ^5^Department of Biotechnology, Maharishi Markandeshwar (deemed to be) University, Mullana, Haryana, India.; ^6^Department of Medicine, Maharishi Markandeshwar (deemed to be) University, Mullana, Haryana, India.; ^7^Department of Pharmacology and Neuroscience, University of North Texas Health Science Center, Fort Worth, Texas, USA.; ^8^Department of Biochemistry and Central Research Cell, Maharishi Markandeshwar (deemed to be) University, Mullana, Haryana, India

**Keywords:** GRP78, *Rhizopus*, rhino-orbito-cerebral, spleen tyrosine kinase

## Abstract

There has been a surge of mucormycosis cases in India in the wake of the second wave of COVID-19 with more than 40000 cases reported. Mucormycosis in patients of COVID-19 in India is at variance to other countries where Aspergillus, Pneumocystis, and Candida have been reported to be the major secondary fungal pathogens. We discuss the probable causes of the mucormycosis epidemic in India. Whereas dysglycaemia and inappropriate steroid use have been widely suggested as tentative reasons, we explore other biological, iatrogenic, and environmental factors. The likelihood of a two-hit pathogenesis remains strong. We propose that COVID-19 itself provides the predisposition to invasive mucormycosis (first hit), through upregulation of GRP78 and downregulation of spleen tyrosine kinase involved in anti-fungal defense, as also through inhibition of CD8+ T-cell mediated immunity. The other iatrogenic and environmental factors may provide the second hit which may have resulted in the surge.

There has been an upsurge of mucormycosis cases in India close on the heels of the second wave of COVID-19. A major portion of cases have been of rhino-orbito-cerebral mucormycosis affecting those who have co-existent COVID-19 or have recently recovered from COVID-19. Mucormycosis is caused by the fungi *Rhizopus sp.*, *Apophysomyces sp., Lichtheimia sp., Mucor sp.*, and others, belonging to the order Mucorales [1]. The invasive disease with high morbidity and more than 50% mortality has been made notifiable and declared an epidemic by several states in India. Dedicated mucormycosis in-patient services have been initiated in major hospitals as more than 40,000 cases have been reported from across the country as of late June 2021. In pre-COVID times, India has been reported to have a high prevalence of mucormycosis of around 140 per million population which is nearly 80 times that observed in developed nations (www.who.int/india/emergencies/coronavirus-disease-(covid-19)/mucormycosis) Major experts in mucormycosis research in India have hypothesized the possible reasons for high occurrence to be abundance of Mucorales in the environment due to a predominant hot and humid climate, and a high prevalence of diabetes in Indians, especially neglected and undiagnosed diabetes [2]. There may also be genetic predisposition to specific fungal types, though this domain still lies unexplored. Despite the prior high prevalence, the post-COVID-19 surge of mucormycosis has been puzzling for healthcare providers and infectious disease specialists.[3] Mucormycosis cases have often been observed in the past in patients with diabetic ketoacidosis and uncontrolled blood glucose levels [4]. Even among COVID-19 patients, most mucormycosis cases are occurring in those with co-existent diabetes. However, the upsurge has been too high to explain, by diabetes alone and many patients do not fit the classic description of poorly controlled diabetes either. Further, unlike other countries where *Aspergillus* and *Candida* have been common fungal pathogens in severe COVID-19 patients, mostly those with a prolonged ICU stay, rampant mucormycosis seems to be unique to India. Searching for other tentative causal factors has thus attained relevance. The factors responsible may be broadly categorised as disease-specific, iatrogenic, and environmental.

COVID-19, like many other viral infections, causes immune dysregulation involving both the innate and the adaptive immune systems. A peripheral lymphopenia with decreased counts of total T cells, CD4+ and CD8+ T cells and natural killer (NK) cells is characteristic of COVID-19; the CD8+ cells are decreased more significantly than CD4+, and both of them show the surface expressions of several T cell exhaustion markers and other functional impairments like decreased proliferation and production of IFN-γ and TNF-α [5-9]. Many factors may contribute to the peripheral lymphopenia in COVID-19, but high circulating levels of cytokines play a crucial role in this process.[6-9] Decreased naïve T-cell response in COVID-19 is further aggravated in males, in old age and in the presence of type 2 diabetes mellitus, enhancing the chances of a cytokine storm[10]. Decreased numbers and impaired functions of dendritic cells (DCs), and NK cells have also been reported in COVID-19 [5, 7, 11, 12]. Circulating monocytes are not decreased in COVID-19, but some of them display abnormal morphology and functional properties especially a mixed M1/M2 phenotype [11, 13]. The anti-fungal immune response is complex and involves the participation of innate immune cells like macrophages and DCs on one hand and on the other hand, the effector cells of adaptive immunity, predominantly CD4+ and partly CD8+ T cells [14-16]. The monocytes, macrophages and DCs through cell-surface pattern-recognition receptors such as C-type lectin like receptors or CLRs (e.g. dectin 1, dectin 2 and others), toll-like receptors (TLRs) and several others, recognise specific residues on fungi (β-glucan or high-mannose oligosaccharide residues on the glycoproteins) and trigger cell signalling pathways that involve two important downstream components, spleen tyrosine kinase or SYK and CARD9 [15-17]. The signalling process leads to increased phagocytosis, production of reactive oxygen species (ROS) and secretion of pro-inflammatory cytokines by the innate immune cells; additionally CD4+ and CD8+ T cells are activated to secrete IL-17, IFN-γ, TNF-α, GM-CSF and others [14-17]. Thus, an anti-fungal response is mounted. NK cells also take part in the antifungal immune response by direct cytotoxic action and through the secretion of cytokines [15]. It is but natural that the decreased numbers and impaired functions of CD4+ T cells, NK cells, DCs and phagocytes as observed in COVID-19 are likely to impair the immune response against fungal invasion. In this connection it is important to point out that urine proteomic analysis of COVID-19 patients has shown a significant downregulation of SYK which is involved in dectin 1 and dectin 2 mediated signalling mechanisms in innate immune cells against fungal infections [18].

Another factor of note, associating COVID-19 and mucormycosis may be related to glucose-regulated protein 78 or GRP78, a chaperone and a member of the heat-shock family of proteins (Hsp70), which helps in protein folding, maturation and assembly and is present in the endoplasmic reticulum (ER) and on the cell surface [19, 20]. The cell surface GRP78 which is translocated from the ER under cellular stress and in various pathological conditions, can bind a variety of ligands and serve as the entry site for viruses like Dengue, Ebola, Zika, Japanese Encephalitis, MERS-CoV and others [19-21]. A recent case-control study has reported an increased expression of GRP78 in COVID-19, and further from experimental studies this receptor has been suggested as an alternative entry point for SARS-CoV-2 [21-23]. On the other hand, the Mucorales are known to invade epithelial and endothelial cells by binding to cell surface GRP78 receptors causing invasive mucormycosis [20, 24]. Though more evidence is needed, there seems to be a clear molecular link between COVID-19 and mucormycosis through GRP78. Hyperglycemia and accumulation of keto-acids have been shown to enhance fungal growth and the expression of GRP78 [24, 25]. Thus, diabetes with or without keto-acidosis is a common co-morbidity which could further enhance GRP78 expression in COVID-19 patients and the consequent incidence of mucormycosis.

Iatrogenic and environmental factors also need consideration. The COVID-19 pandemic has been characterised by rapidly changing therapeutic guidelines, use of novel medications with often a narrow evidence-base, and often irrational polypharmacy [26]. Steroids gained prominence in COVID-19 therapeutics with the Recovery trial [27]. However, there has been rampant use of steroids in high doses, for prolonged periods and often without clear indication, in India. Much of this has been due to the misadventures of rural quacks but some steroid overuse can be attributed to trained doctors in resource-limited settings too. Besides being immune suppressants, steroids in higher doses have been notorious in causing dysglycaemia which may promote mucormycosis. The use of medications such as the interleukin-6 inhibitor tocilizumab and the RNA polymerase inhibitor remdesivir have often been in desperation and out of fear of adverse prognosis, as hospitals were overwhelmed in the second wave. Tocilizumab has shown modest mortality benefits in COVID-19 patients with hypoxia and critically ill patients requiring respiratory support in open-label controlled settings [28, 29]. Mortality benefits by tocilizumab have also been emphasized in various pooled analyses of observational studies [30, 31]. The drug however is known to cause immune suppression, neutropenia, and secondary infections [32]. IL-6 and IL-6 receptor knock out animal models have displayed reduced neutrophilic response against fungal infections [33]. The extent to which blockade of IL-6 signalling could contribute to the surge of mucormycosis remains however to be definitely addressed. With regard to the therapeutic role of remdesivir in COVID-19, the evidence is conflicting so far and a conditional recommendation against the use of remdesivir had been issued by the WHO (www.who.int/news-room/feature-stories/detail/who-recommends-against-the-use-of-remdesivir-in-covid-19-patients). The authors have observed neutropenia to develop with remdesivir use too. A potentially interesting point is the overuse of two apparently benign agents, vitamin C and zinc in excess, in COVID-19 management protocols in India. Whereas both the agents may have doubtful beneficial role in viral fever syndromes, high doses may be detrimental [34]. Vitamin C has been prescribed at doses up to 1500 mg/day over prolonged periods and may lead to a lowering of blood pH especially in COVID-19 patients with compromised renal functions. Interestingly, acidosis leads to over-expression of GRP78 in primary human dermal microvascular endothelial cells (HDMEC) [35]. Further, vitamin C promotes intestinal iron absorption, iron being a promoter of Mucorales growth. Zinc, which has also been used for prolonged periods without definite indication, has been connected in the past with fungal virulence. Zinc is essential for fungal growth, with many zinc-binding proteins involved in transcription regulation, sugar and amino acid metabolism, cell division, and nitrogen utilisation. Further, zinc-containing superoxide dismutases (SODs) and metalloproteases, are an integral part of fungal virulence. SODs form the major fungal defence against reactive oxygen species generated by host immune cells. Being a trace element, the requirement for zinc is low in humans. Supplementing zinc in high doses for prolonged periods may serve to stimulate fungal growth [36].

The role of environmental and social factors is more debatable and current evidence is limited. Repeated use of unclean masks and improper mask hygiene, especially among the poor, inadequate sterile practices in storage and use of nasal/pharyngeal swabs for SARS-CoV-2 detection may be potential contributors. The use of industrial oxygen and irregular cleaning of humidifiers in oxygen delivery systems in over-burdened hospitals have been suggested to have a role in the mucormycosis-epidemic. There has been an increase in the use of traditional Ayurvedic medications and oils for nasal instillation. Many of these have been sold as over the counter products without proper quality checks and without due prescription by trained Ayurvedic physicians. Contamination of such products may also be a source of concern.

A noteworthy aspect is that unlike the second wave, the first wave of COVID-19 in India was not associated with a surge of mucormycosis. Though a definitive explanation is not available, several possibilities could be mentioned. Firstly, absolute numbers of COVID-19 cases during the second wave were much higher than in the first wave, which was also flatter and of longer duration than the second wave (www.worldometers.info/coronavirus/country/india).Thus, mucormycosis cases during the first wave might have been much less and sparsely distributed over a prolonged time interval. Because mucormycosis is an infrequent complication of COVID-19, the cases during the first wave were probably masked by the non-COVID-19 mucormycosis which has been generally prevalent in India. There exists some evidence of mucormycosis occurring during the first wave too with cases reported as early as in October-November 2020 [37]. Secondly, the second wave of COVID-19 overwhelmed the limited health care facilities in large parts of India, and the situation increased the risk of nosocomial infection from unhygienic and contaminated health care and life-support devices in many overcrowded hospital wards. Lastly, multiple ‘variants of concern’ of SARS-CoV-2 emerged during the pandemic across the globe as a result of mutations in both the spike and non-structural proteins of the virus leading to altered viral characteristics; several such variants (B.1.1.7, B.1.617, B.1.351, B.1.618, B.1.1.28.1 etc.) were reported from India during the second wave and these variants might have a stronger dys-regulatory effect on the adaptive immune response of the body, making the host vulnerable to opportunistic infections [38-41]. This last possibility opens up new avenues of research in COVID-19.

Each of the discussed factors may or may not have aided in the causation of mucormycosis. We believe a two-hit hypothesis remains the most likely, the first hit being provided by COVID-19 and the second by one or more of the other factors discussed as well as individual-specific determinants which may have genetic components. Identifying the reasons for the mucormycosis surge through targeted basic research would be an enthralling affair.
